# Organic and Inorganic Mercury in Neonatal Rat Brain after Prenatal Exposure to Methylmercury and Mercury Vapor

**DOI:** 10.1289/ehp.0900956

**Published:** 2009-09-29

**Authors:** Hiromi Ishitobi, Sander Stern, Sally W. Thurston, Grazyna Zareba, Margaret Langdon, Robert Gelein, Bernard Weiss

**Affiliations:** 1 Department of Environmental Medicine and; 2 Department of Biostatistics and Computational Biology, School of Medicine and Dentistry, University of Rochester, Rochester, New York, USA

**Keywords:** brain, coexposure, inorganic mercury, mercury vapor, methylmercury, organic mercury, prenatal

## Abstract

**Background:**

Many populations are exposed to multiple species of mercury (Hg), predominantly organic Hg as methylmercury (MeHg) from fish, and inorganic Hg as Hg vapor from dental amalgams. Most of our knowledge of the neurotoxicity of Hg is based on research devoted to studying only one form at a time, mostly MeHg.

**Objectives:**

In this study we investigated the effects of prenatal exposure to MeHg and Hg vapor on Hg concentrations in the brain of neonatal rats.

**Methods:**

Female Long-Evans hooded rats were exposed to MeHg (0, 3, 6, or 9 ppm as drinking solution), Hg vapor (0, 300, or 1,000 μg/m^3^ for 2 hr/day), or the combination of both, from 30 days before breeding through gestational day 18. On postnatal day 4, whole brains were taken from one male and one female from each of four litters in each treatment group to assess organic and inorganic Hg in the brain by cold vapor atomic absorption spectrometry.

**Results:**

Statistical analysis using linear mixed effects models showed that MeHg dose was the primary determinant of both organic and inorganic brain Hg levels. For both outcomes, we also found significant interactions between MeHg and Hg vapor exposure. These interactions were driven by the fact that among animals not exposed to MeHg, animals exposed to Hg vapor had significantly greater organic and inorganic brain Hg levels than did unexposed animals.

**Conclusion:**

This interaction, heretofore not reported, suggests that coexposure to MeHg and Hg vapor at levels relevant to human exposure might elevate neurotoxic risks.

Mercury (Hg) toxicity is the focus of substantial public concern. Pronouncements about the safety of fish in the diet and about adverse health effects attributable to dental amalgams are the primary bases underlying this concern. Hg occurs in different forms in the environment; Hg species are classified as elemental (Hg^0^), inorganic (Hg^2+^, Hg^+^), and organic [e.g., methylmercury (MeHg)]. However, many populations are exposed to more than one form. Almost all of our knowledge is based on research devoted to only one Hg species. How different species of Hg act in combination remains unclear, which leads to significant gaps in our understanding of Hg toxicity.

MeHg, to which humans are primarily exposed through consumption of contaminated fish, shellfish, and sea mammals [U.S. Environmental Protection Agency (EPA) 1997], is a potent neurotoxicant to both the mature and developing central nervous system. Several studies have shown potential adverse effects on child development from prenatal exposure to MeHg (for review, see Clarkson and Magos 2006). Observations in human populations demonstrate that MeHg readily crosses the placental barrier, as indicated by cord blood levels higher than those seen in maternal blood (Vahter et al 2000). MeHg can penetrate into the fetal brain (Aschner and Aschner 1990; Choi et al. 1978; Davis et al. 1994; Day et al. 2005; Newland et al. 2006; Newland and Reile 1999; Stern et al. 2001), which allows for accumulation in the central nervous system. Because MeHg is slowly converted to inorganic Hg in brain tissue, and as such resides there for many years (Charleston et al. 1995; Davis et al. 1994), the relative contribution of the intact organomercurial versus the inorganic metabolite to neuronal damage remains an open question (Magos 1987), with significant implications for risk assessment.

Another major source of Hg exposure is inorganic Hg in the form of Hg vapor. Exposure of the general population to Hg vapor occurs primarily through inhalation of Hg vapor released from dental amalgams [Brownawell et al. 2005; International Programme on Chemical Safety (IPCS) 1991]. Although the levels from such exposures are considered “low” with respect to some known health effects, we know little about how those exposures might interact with MeHg toxicity. Approximately 80% of inhaled Hg vapor is retained and absorbed in blood during pulmonary circulation (Hursh et al. 1976). Although Hg vapor introduced into the body is trapped and oxidized to divalent inorganic Hg in erythrocytes, part of the Hg vapor remains in the bloodstream long enough for it to be distributed to other tissues and reach the blood–brain barrier (Yoshida 2002). Non-ionized Hg readily penetrates the placental barrier (Clarkson et al. 1972; Khayat and Dencker 1982) and is taken up by fetal tissues, including brain. The ability of inhaled Hg vapor to accumulate in the fetal brain has also been shown in human and animal studies (Drasch et al. 1994; Morgan et al. 2002, 2006). Inhaled Hg vapor has been known to damage the adult central nervous system (Echeverria et al. 1998; Goldwater 1972). On the other hand, only a limited number of studies have shown behavioral changes in animals prenatally exposed to Hg vapor (Danielsson et al. 1993; Fredriksson et al. 1996). However, the study designs of these experiments were not relevant to human exposure because of rather high concentrations of Hg vapor and short durations of exposure.

Although studies of combined exposures are limited, we do know that both agents *a*) produce prenatal damage; *b*) produce behavioral changes in animals at levels that do not produce clinically apparent toxicity; and *c*) result in deposition in the brain of the same toxic species of Hg, namely, mercuric Hg. Yet there are reasons to suggest that combined exposure to MeHg and inhaled Hg vapor might produce effects different from those seen from exposure to either agent alone:

- MeHg is well known to affect the developing central nervous system, whereas effects of inhaled Hg vapor on that system are not clear.
- Metabolism of MeHg differs from that of Hg vapor (Clarkson and Magos 2006).
- Brain pathology and the signs and symptoms of MeHg poisoning differ from those of inorganic Hg poisoning (Clarkson and Magos 2006).
- Although both MeHg and Hg vapor result in deposition in the brain as mercuric Hg, divalent inorganic Hg (Hg^2+^) is believed to be the proximate toxic agent in the case of poisoning from inhaled Hg vapor, but this does not appear to be the case for MeHg (Clarkson and Magos 2006).
- Delayed manifestation of the effects of MeHg is seen in humans and animals (typical examples of latent toxicity in humans, including both acute and chronic MeHg exposures have been described) (Weiss et al. 2005a), and latent toxicities in animals have been shown (Newland and Rasmussen 2000; Newland et al. 2004; Rice 1996; Yoshida et al. 2008), but these are not known for inorganic vapor.

Even so, despite the considerable public health implications of coexposure, only Fredriksson et al. (1996) have shown that exposure to MeHg can further worsen adverse behavioral performance compared with inhaled Hg vapor alone. In their experiment, rat dams were exposed to Hg vapor [1,800 μg Hg/m^3^ for 1.5 hr/day from gestational day (GD) 14 to GD19), MeHg (2 mg/kg/day from GD6 to GD9], or both.

We designed the following experiments to broaden our understanding of the effects of joint exposure to Hg vapor and MeHg, a situation that more realistically models human exposures, which are both concurrent and chronic and generally begin long before pregnancy.

## Materials and Methods

### Subjects

We conducted two experiments (experiments 1 and 2); we used the preliminary analysis of experiment 1 to select the Hg doses for experiment 2. The basic experimental schemes were the same between the two experiments except for the Hg doses. For the experiments, we used 99 (experiment 1) and 96 (experiment 2) female Long-Evans rats (Harlan, Indianapolis, IN) 8–9 weeks of age when they arrived at the University of Rochester Medical Center Vivarium (a facility certified by the Association for Assessment and Accreditation of Laboratory Animal Care). Animals were housed individually, in rooms maintained at 23 ± 2°C with a 12-hr light–dark cycle with light onset at 0600 hours, in polycarbonate breeder cages with wire covers and filter tops. The females were allowed free access to Teklad Global 2018 Rodent Diet (Harlan), which does not contain fish meal or animal protein, and drinking water solutions (described below) except when the rats were in the exposure chambers. We chose that diet to minimize uncontrolled Hg contamination (Weiss et al. 2005b). The rats were randomly assigned to groups as shown in Table 1. Exposure chamber space and exposure durations limited the number of groups per experiment, so the 6-ppm MeHg × 300 μg/m^3^ Hg vapor group was excluded in experiment 1. Long-Evans male rats (Harlan), 75 retired breeders in experiment 1 and 80 rats 8–9 weeks of age in experiment 2, were received 3 weeks after the females and housed under the same conditions and in the same rooms as the females.

All animals were treated humanely and with regard for alleviation of suffering. All were monitored daily by the research staff and personnel from the Division of Laboratory Animal Medicine of the University of Rochester Medical Center. All experimental procedures were approved by the University Committee on Animal Research.

### Exposure to Hg

Groups of rats were exposed to *a*) MeHg as MeHg chloride via drinking water, *b*) Hg vapor, or *c*) both MeHg and Hg vapor; *d*) a separate group was given Na_2_CO_3_ vehicle (controls). Exposures occurred daily for 30 days before breeding to attain a stable Hg burden in the females so as to simulate the human exposure pattern. Exposures continued until GD18.

### Hg vapor

Before beginning the exposures, the females were adapted for 1 week to the routine of being transported from their home quarters to the inhalation facility and placed in the exposure chambers. This was done to preclude excessive uptake of Hg during initial exposures, which would be expected in the absence of adaptation (Stern et al. 1996). The 2-hr exposure of female rats to Hg vapor was conducted in two adjacent hexagonal Rochester chambers (one control, one exposure), each having an internal capacity of 2 m^3^ (Chen and Moss 1989). The chambers were supplied with filtered and conditioned outside air drawn into the chamber through an intake duct at the top and discharged into an exhaust manifold with an Hg-trapping filter at the bottom. Chamber temperatures were maintained at 22–24°C. Metallic Hg was heated in a flask located adjacent to the exposure chamber. Vapor concentrations were controlled by adjusting the flow of heated air passing over the Hg, which was then mixed with the main airstream flowing into the chamber. During exposure, the Hg vapor concentration was monitored continuously with an ultraviolet Hg monitor. The calibration of the monitor was confirmed using a Jerome 431-X mercury vapor analyzer (Arizona Instrument LLC, Chandler, AZ), which was certified by an independent testing laboratory. The output was connected to a desktop computer for both online monitoring and temporary storage of sampling data files. For exposures, the subjects were held individually in open-mesh metal cages. The 2-hr session was designated as beginning when the Hg concentration reached 66% of the target concentration (~ 10 min; it reached 90% of the target value in about an additional 1–2 min). Once attained, the concentration was maintained at the target value (± 5%). At the end of the scheduled exposure, the Hg-vapor generator was turned off, and the airflow over the Hg was stopped. Chamber concentration declined rapidly, falling to ≤ 30 μg/m^3^ in just a few minutes. The females were then removed after another 30 min in order to completely exhaust the chambers. Exposure continued from 30 days before breeding through GD18.

### MeHg

The females were dosed with an MeHg chloride (CH_3_HgCl) drinking solution 30 days before breeding through GD18. For dosing, we prepared 100 ppm MeHg stock solution weekly by dissolving crystalline CH_3_HgCl (Alfa Aesar, Ward Hill, MA) in a buffer solution of 5 mM sodium carbonate (Na_2_CO_3_; Mallinckrodt & Baker, Inc., Phillipsburg, NJ). The stock solution was diluted to produce requisite quantities of the dosing solutions. The Na_2_CO_3_ solution was also used for the 0-ppm control group. Solutions were stored in glass bottles with neoprene stoppers and stainless steel spouts.

MeHg concentration was confirmed by cold vapor atomic absorption spectrometry and found to be within 5% of the target value.

### Breeding and litters

After 30 days of exposure, individual females were randomly placed with the males at 1600–1700 hours. The female’s drinking solution was always kept on the cage in which she was located, so it was on the breeding cage only when she was physically present with the male. Vaginal smears were obtained at 0600 hours and examined microscopically for the presence of sperm, and the female was then returned to her home cage. The day a sperm-positive smear was observed was defined as GD0. A male was paired with the same female(s) until a sperm-positive smear was observed, or after three successive nights, whichever came first. Some males were paired with a second female, which was always a member of a different exposure group. Immediately after the Hg vapor exposure on GD18, blood was drawn from the tail of four females in each group into heparinized calibrated micropipets (Drummond Scientific Co., Broomall, PA). Blood samples were stored in 0.9% NaCl (Sigma-Aldrich, St. Louis, MO) at 4°C until assayed.

The day on which a litter was discovered (up to 1300 hours) was designated as postnatal day (PND) 1. Litter size and sex, body weight, and overall health status of each pup were checked and recorded.

On PND4, litters were culled via random sampling to three pups of each sex per litter in experiment 1 and four in experiment 2. For culling, pups were separated by sex and then separated, held individually in a fixed order, and weighed. A pup was culled immediately if its weight fell below 75% of the mean for that sex in that litter, unless needed for brain sampling. Then, after a list of random numbers equal to the number of remaining pups for that litter/sex, pups were kept or culled.

Brains were sampled on PND4 from one male and one female littermate from each of four litters in each group. Some brains were collected from pups weighing < 75% of the mean for that sex in that litter. Pups were sacrificed after carbon dioxide anesthesia, and the whole brain was removed and weighed. The brains were stored at −12°C until assayed.

### Hg assays

We determined Hg levels in brain and blood by cold vapor atomic absorption spectrophotometry using a flameless atomic absorption monitor (model 1235; Laboratory Data Control, Riviera Beach, FL). All details of analysis and sample preparation have been previously described (Magos 1971; Magos and Clarkson 1972). The method is based on the rapid conversions of Hg compounds into atomic Hg. Cadmium chloride/stannous chloride reagent is used to reduce total Hg (organic plus inorganic), whereas stannous chloride selectively reduces inorganic Hg. Organic Hg is the difference between total and inorganic Hg. We determined Hg in brain after digestion with 40% sodium hydroxide, and in blood after sample dilution with saline. For standards preparation, we used Mercury Reference Standard Solution (SM 114-100; Fisher Scientific, Fairmont, NJ). Detection limits (LODs) and quantification limits were calculated from blank measurements following the recommendations of the International Union of Pure and Applied Chemistry (Currie 1999). The LODs (3 × SD for blanks) were 19.5 ng/g for total Hg, 9.75 ng/g for inorganic Hg for brain, and 11.5 μg/L for both total and inorganic Hg for blood, whereas limits of quantification were 65.0 ng/g, 32.5 ng/g, and 38.3 μg/L, respectively. For samples with values < LOD, we used one-half the LOD for statistical analysis. The method imprecision, calculated as the coefficient of variation for duplicate preparations measurements, was 4%. We evaluated the analytical accuracy of Hg determination using reference material (certified human blood samples from the Centre de Toxicologie du Quebec, International Comparison Program, Quebec, Canada). Our result was 76.65 ± 1.41 ng/g for lot M-08-14, compared with 79 ng/g (range, 58–100 ng/g) the results from the Centre de Toxicologie du Quebec. Participation in external quality control programs also rendered highly satisfactory data.

### Statistical analysis

#### Breeding outcomes and Hg in dam’s blood

For breeding outcomes, we used the litter as the unit for analysis, and for dam’s blood, we used the dam. We evaluated outcomes (of the averaged parameters if needed for the breeding outcomes) using one-way analysis of variance (ANOVA) to determine differences among groups (defined by Hg vapor and MeHg concentrations), and using two-way ANOVA to determine the effects of MeHg, Hg vapor, and their interaction. Values of *p* < 0.05 were considered statistically significant. We used a logarithmic (base *e*) transformation of many outcomes in order to satisfy model assumptions.

#### Models for pup’s brain Hg

We applied linear mixed-effects models (McCulloch and Searle 2001) using R software (Faraway 2006; see also R Foundation for Statistical Computing 2009) to examine the relations among MeHg dose, Hg vapor dose, sex, experiment, and two outcomes: organic and inorganic Hg brain levels. The two outcomes were examined in separate models. Data from experiments 1 and 2 were combined for both outcomes. The inclusion of the experiment term in the model allows the two experiments to have different intercepts. The mixed model includes a random litter effect, which models the correlation between pups within a litter and allows the pup to be the unit of analysis. This general approach was also used by Gray et al. (2009).

We started by treating both MeHg dose and Hg vapor dose as continuous variables; when the linearity assumption held, we treated them as continuous variables in the final model. For both outcomes we started by considering a full model, which also included all two-way and three-way interactions among Hg vapor dose, MeHg dose, and sex. When the three-way interaction was not significant, we fit a model without this term. We then considered models without two-way interactions and without main and/or covariate effects, when these terms were not significant.

To satisfy model assumptions, it was necessary to use a logarithmic (base *e*) transformation of each Hg brain level outcome. For both outcomes there was no difference in response between low and high Hg vapor dose, so this variable could be collapsed into two categories: no versus any Hg vapor exposure. Neither sex of the pup nor experiment was a significant predictor for either outcome, indicating in part that data from both experiments could be combined into a single model. For organic Hg brain levels, we found a linear relationship between the logarithm of organic Hg brain levels and the logarithm of MeHg dose (after adding 0.l to avoid taking the logarithm of 0), and our final model included three terms: log(MeHg dose + 0.1), any Hg vapor exposure, and their interaction. The interaction allows the slope relating MeHg dose to organic brain Hg outcome to differ for Hg vapor–exposed compared with non-Hg vapor–exposed pups.

For inorganic Hg brain levels, the relationship to MeHg dose was not quite linear. Our final model for this outcome included three terms or groups of terms: three indicator variables for MeHg dose, any Hg vapor exposure, and their interactions. Sex and experiment were not significant predictors.

## Results

### Breeding outcomes

The number of litters and number of pups per litter in each group on PND1 and PND4 are shown in Tables 2 and 3. On PND1, the groups in experiments 1 and 2 did not differ in litter size (the group average varied from 8.7 to 11.9 pups in experiment 1 and from 8.1 to 11.5 in experiment 2) or in sex ratio within a litter (1.07–1.58 and 0.97–2.08 in experiments 1 and 2, respectively). Body weights on PND1 did not differ. The number of pups in the 6- and 9-ppm MeHg dose groups decreased between PND1 and PND4 in experiment 2. Two-way ANOVA showed that MeHg had a main effect on body weight of both male (*p* = 0.0203) and female (*p* = 0.0055) pups on PND4 in experiment 2 but not in experiment 1. We found no effects of Hg vapor and interaction of MeHg and Hg vapor.

### Hg in dam blood

**Figure 1:**
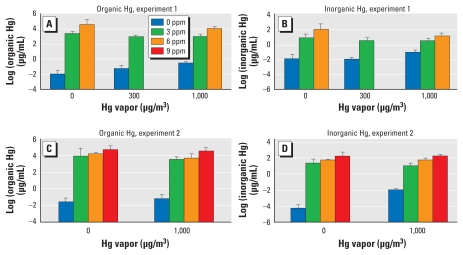
Concentrations (mean ± SD) of organic Hg (*A* and *C*) and inorganic Hg (*B* and *D*) in dam blood on GD18 from experiment 1 (*A* and *B*) and experiment 2 (*C* and *D*).

We compared the logarithm of Hg levels (total, inorganic, and organic) in blood on GD18 across groups, separately by experiment, as shown in Figure 1. We found significant differences among the groups (*p* < 0.0001 for total, inorganic, and organic Hg) in both experiments. Two-way ANOVA with interactions showed that in experiment 1, the interactions between MeHg and Hg vapor were significant for all three outcomes (*p* = 0.0006 for total Hg, *p* = 0.002 for inorganic Hg, and *p* = 0.0002 for organic Hg, each for a 3 df test). In experiment 2, the interactions were significant for inorganic Hg (*p* < 0.0001) but not for total or organic Hg. MeHg was a very strong predictor of total, inorganic, and organic Hg in both experiments (*p* < 0.001). With the exception of inorganic Hg in experiment 2, Hg vapor did not affect Hg levels.

### Hg in pup brain

**Figure 2:**
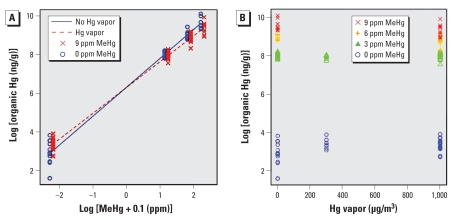
Organic Hg in pup brain on PND4 by log scale of MeHg (*A*) and absolute value of Hg vapor (*B*). Data points show individual pups. The fitted values in (*A*) exhibit a clear linear relationship between the logarithm of organic Hg brain levels and the logarithm of MeHg dose. The interaction allows the slope relating MeHg dose to organic brain Hg outcome to differ for Hg vapor–exposed compared with non-Hg vapor–exposed pups. Interaction of MeHg and Hg vapor on organic Hg values, *p* < 0.0001; organic brain Hg is strongly predicted by MeHg dose.

Total, inorganic, and organic Hg levels in pup brains for each group are summarized in Tables 4 and 5. Figure 2 shows the observed (points) and fitted values (lines) of organic Hg in pup brain, where both the brain levels and the MeHg dose are on the logarithmic scale. The vapor and no-vapor points are offset slightly in Figure 2A for clarity. We found an interaction of MeHg and Hg vapor on organic brain Hg (*p* < 0.0001; Figure 2A). Among animals exposed to Hg vapor, the predicted levels of brain organic Hg were larger among animals not exposed to MeHg, but organic Hg levels rose less steeply with increasing MeHg exposure compared with those in animals not exposed to Hg vapor. Figure 2B shows the relationship between organic brain Hg and Hg vapor, separately by MeHg concentration. As Figure 2 illustrates, organic brain Hg was very strongly predicted by MeHg dose.

**Figure 3:**
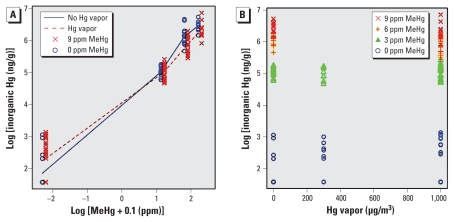
Inorganic Hg in pup brain on PND4 by log scale of MeHg (*A*) and absolute value of Hg vapor (*B*). Data points show individual pups. The fitted values are shown in (*A*). For the interaction between Hg vapor and the MeHg dose indicator variables, *p* = 0.02 for the 3 df test. MeHg was a very strong predictor of inorganic brain levels (*p* < 0.001 for each MeHg dose).

MeHg was also a very strong predictor of inorganic brain levels (*p* < 0.001 for each MeHg dose; Figure 3A). The observed (points) and fitted values (lines) of inorganic Hg in pup brains are shown in Figure 3. For inorganic Hg brain levels, we coded MeHg dose with three indicator variables to distinguish among the four MeHg dose levels. This analysis showed interactions between Hg vapor and the MeHg dose indicator variables (*p* = 0.02 for the 3 df test).

**Figure 4:**
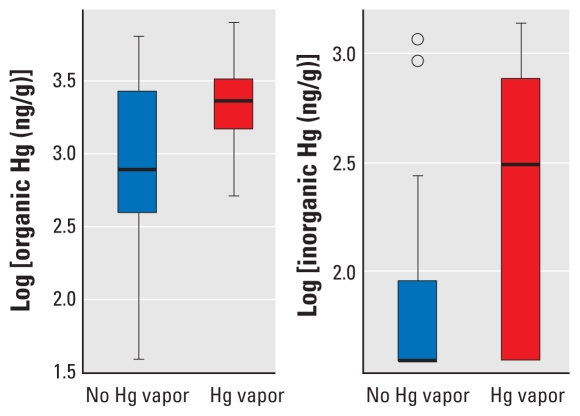
Box plots of log scale brain concentrations on PND4 for organic Hg (*A*) and inorganic Hg (*B*) for pups exposed to no Hg versus pups exposed to any Hg vapor. The solid line within the box shows the median; the top and bottom of the boxes are the 75th and 25th percentiles, respectively; and the whiskers extend to the largest or smallest observation that is within 1.5 times the length of the box.

In summary, the dose of MeHg drove levels of both organic and inorganic Hg in pup brains. Exposure to Hg vapor lowered pup brain Hg levels at high MeHg doses and increased them at low MeHg doses compared with animals not exposed to Hg vapor. Separate analysis showed that among the 40 animals with no MeHg exposure, exposure to any Hg vapor (*n* = 24 animals) was associated with a higher brain Hg level compared with no exposure to Hg vapor (*n* = 16 animals; *p* = 0.02 for both organic and inorganic Hg; Figure 4).

## Discussion

Prenatal exposure to the combination of MeHg and Hg vapor had interactive effects on the levels of organic and inorganic Hg in rat neonatal brain. Surprisingly, Hg levels were increased by Hg vapor at low MeHg doses, a finding relevant to human exposures, which typically occur at low concentrations.

Total Hg concentration in brains of pups exposed to 6 ppm MeHg without Hg vapor is comparable to that on PND1 in previous studies, in which MeHg dose and duration of exposure were similar to ours (Day et al. 2005; Newland et al. 2006; Newland and Reile 1999). In these studies, rat dams were exposed daily to MeHg for 30 days or longer before breeding, simulating the predominant human exposure pattern of a stable diet. Although we did not determine whether steady-state levels had been attained in the pregnant females, Newland and Reile (1999) found no differences in brain Hg levels of PND0 offspring of females that had been exposed for 28 or 45 days before breeding. Baseline Hg concentrations achieved by these studies are consistent over time, and thus are preferable to short-term exposures as a basis for risk assessments.

Pup brain Hg (total, inorganic, and organic) concentrations increased with the dose of MeHg, but this increase was not linear across the exposure groups. That is, total brain and organic Hg concentrations in pups exposed to 6 or 9 ppm MeHg were greater than two or three times those of pups exposed to the lowest MeHg dose (3 ppm). Newland and Reile (1999) also found that total Hg in pup brain at birth increased nonlinearly with the concentration of MeHg (0.5 or 6.4 ppm in drinking water) when rat dams were exposed to MeHg beginning 28 or 49 days before breeding and through gestation, similar to the present study design. Such nonlinearities have also been seen in nonhuman primates (Lushei et al. 1977). The present data suggest that extrapolation from high-concentration exposures may distort estimates of brain Hg levels at lower MeHg doses. The present data also suggest that with a logarithmic transformation of both pup brain Hg and MeHg dose, a linear dose–response relationship may be reasonable. The increase in inorganic Hg associated with increased MeHg doses was not similar to that measured by organic Hg levels. The increase seen in total and organic Hg as MeHg dose increased was higher between 6 and 9 ppm MeHg than between 0 and 3 ppm or between 3 and 6 ppm, whereas the increase of inorganic Hg was lower. This complex pattern indicates that attempts to estimate brain levels of inorganic Hg on the basis of dose requires that experiments rely on the lower MeHg exposure levels that are relevant to human exposure levels.

In these experiments, inorganic Hg in pup brain increased with increasing MeHg dose. This result is ascribed to the process by which MeHg is converted to the inorganic form (Clarkson 1997; Clarkson and Magos 2006). Moreover, elevated levels of inorganic Hg have been found in the brains of humans and monkeys exposed to MeHg (Davis et al. 1994; Vahter et al. 1995). One site for the process would be phagocytic cells present in many mammalian tissues, including the brain, that are capable of breaking the carbon–Hg bond (Suda and Takahashi 1990). Thus, inorganic Hg in brain tissue may arise from *in situ* metabolism of MeHg. However, it is still possible that some of the inorganic Hg does not represent the *in situ* conversion of MeHg but instead is derived from some distant source via the vapor pathway. Intestinal microflora are also capable of cleaving the carbon–Hg bond (Rowland et al. 1987). Because the vapor produced by reduction of inorganic Hg in the intestine or phagocytic cells in the liver readily crosses the blood–brain barrier to be oxidized in brain tissue (Clarkson 1997), some portion of the inorganic Hg in pup brain may arise from this process. However, it is not clear whether it occurs in the fetus or in the mother. Because MeHg readily crosses the placental barrier (Vahter et al. 2000), MeHg transferred from mother to fetus may be converted to inorganic Hg in the fetus. Also, inorganic Hg converted from MeHg in the mother may be transferred to the fetus via placenta and reach fetal brain as non-ionized Hg, which also crosses the placental and blood–brain barrier (Clarkson et al. 1972; Khayat and Dencker 1982; Yoshida 2002). Contributions to pup brain inorganic Hg may differ between fetus and mother.

Exposure to Hg vapor increased brain Hg levels (total, inorganic, and organic) in the pups not exposed to MeHg. The increase in brain Hg was not related to the concentration of Hg vapor, which may suggest that the Hg level in the pup brains reached steady state before the time of brain sampling. However, Morgan et al. (2002, 2006) showed that, in the brains of neonates perinatally exposed to Hg vapor (1, 2, 4, or 8 mg/m^3^ for 2 hr/day during GD6–GD15), total Hg concentrations increased with increasing exposure dose. The exposure concentrations they employed were higher, and the duration shorter, than those in the present study, which might contribute to the differing results. Although data on the elimination of inorganic Hg in the fetus and/or neonate after gestational exposure are sparse, factors of retention and/or elimination should be considered to evaluate the effect of prenatal exposure to Hg vapor on the brain Hg levels because of the time lag (6–7 days) between the last exposure and the brain sampling. There is unlikely to be any loss of inorganic Hg due to this process, because the methylation of inorganic Hg does not appear to take place to any significant extent in either human or animal tissues (Clarkson and Magos 2006).

So far, only one study has addressed prenatal coexposure to MeHg and Hg vapor (Fredriksson et al. 1996). That study showed that brains of rat offspring (on PND3) prenatally exposed to both MeHg and Hg vapor contained more total Hg than those exposed to either form alone. Statistical analyses of the joint and single contributions of MeHg and Hg vapor to total Hg seem not to have been performed, although coexposure resulted in slightly higher Hg levels in the brain (12 ng/g) than what would had been expected, considering the concentrations obtained after exposure to either MeHg (4 ng/g) or Hg vapor (5 ng/g) alone. The present data suggest that coexposure to Hg vapor slightly lowered brain Hg levels at high MeHg doses and increased them at low MeHg doses. In the study by Fredriksson et al. (1996), the MeHg dose was 2 mg/kg/day, which is higher than our highest dose, which would be approximately 700–750 μg/kg/day if we extrapolate from Newland and Reile (1999). Similarly, Fredriksson et al.’s dose of Hg vapor was 1.8 mg/m^3^ for 1.5 hr/day, which is also higher than our highest dose (1.0 mg/m^3^ for 2 hr/day). In addition, MeHg exposure occurred only during GD6–GD9, and that of Hg vapor occurred during GD14–GD19, which means that the two exposures did not occur simultaneously. Different doses and duration of exposures might explain the different outcomes.

Several metabolic processes could account for the observation that exposure to Hg vapor lowered brain Hg levels at high MeHg doses. Inorganic Hg, but not MeHg, can induce the metal-binding protein metallothionein. Binding to this protein is generally regarded as a detoxication process (Clarkson 1997; Clarkson and Magos 2006) and has been proven to play an important role in the retention of Hg in tissue. Metallothionein induced by Hg vapor in the mother and/or in the fetus might prevent MeHg and perhaps inorganic Hg from reaching the fetal brain. The conversion of MeHg to inorganic Hg may also need to be considered (Clarkson 1997; Clarkson and Magos 2006). Inorganic Hg in the form of oxidized Hg has a limited capacity to cross the blood–brain and placental barriers (Clarkson 1997; Clarkson and Magos 2006). The presence of large amounts of Hg vapor might promote the oxidation of MeHg in the mother and/or in the fetus, resulting in less Hg reaching the brain. However, this hypothesis has not yet been tested.

The main source of human exposure to MeHg is the diet, especially fish and seafood (U.S. EPA 1997). Dietary intake of MeHg is estimated at 0.1–2.0 μg/kg body weight per week for numerous national diets (IPCS 2004). The U.S. EPA’s current reference dose for MeHg is 0.1 μg/kg body weight/day (U.S. EPA 2001 2009). The 53rd meeting of the Joint Food and Agriculture Organization/World Health Organization Expert Committee on Food Additives established provisional tolerance weekly intake of 200 μg MeHg (3.3 μg/kg body weight) for the general population but noted that fetuses and infants might be more sensitive than adults to its toxic effects (IPCS 2000). Because there has been no definitive separation of prenatal and postnatal exposure that would permit dose–response modeling, there are currently no data that would support the derivation of a child (vs. general population) reference dose (U.S. EPA 2001). Those values are relatively low compared with doses we used in rats in the present study. Rat blood has approximately 10 times as much hemoglobin as does mouse, monkey, or human blood (Magos 1987) and binds Hg. That means that rat blood has a higher capacity to bind Hg, so a higher intake is required to compare neonatal brain Hg, a better biomarker, across species (Burbacher et al. 1990). However, the present study showed that, at lower MeHg doses, exposure to Hg vapor increased both brain organic and inorganic Hg levels. In addition, the increase in brain Hg did not depend on the Hg vapor dose. That means that brain Hg levels might be higher than expected even if MeHg intake is lower than the provisional tolerance weekly intake or U.S. EPA reference dose when fetuses are simultaneously exposed to Hg vapor even at levels as low as those attributable to dental amalgams. This might be one mechanism by which coexposure to dietary MeHg and Hg vapor at levels relevant to human exposure elevates neurotoxic risks and may need to be taken into account for risk assessment calculations. Additional research is required to directly evaluate such outcomes.

## Conclusions

Our study has revealed interactive effects of joint exposure to MeHg and Hg vapor during the prenatal period on organic and inorganic Hg levels in pup brain. Hg vapor increased both forms of Hg in pup brain at lower MeHg concentrations, an outcome relevant to human exposure. Human fetuses exposed to both MeHg and Hg vapor may have increased risks of neurodevelopmental toxicity in contrast to either type of Hg alone.

## Correction

In the manuscript originally published online, right-hand columns showing higher Hg vapor doses were inadvertently omitted from Tables 2–5, error bars were omitted from Figure 1A and 1B, and Figure 2B was incorrect. The tables and figures have been corrected here.

## Figures and Tables

**Table 1:** Group assignment in the two experiments.

	MeHg (ppm)			
Hg vapor (μg/m^3^)	0	3	6	9
Experiment 1				
0	15^a^	12^a^	12^a^	
300	12	12		
1,000	12^a^	12^a^	12^a^	
Experiment 2				
0	12^a^	12^a^	12^a^	12
1,000	12^a^	12^a^	12^a^	12

Values shown are the number of females assigned to each group at the beginning of the experiments. In experiment 1, the 6-ppm MeHg × 300 μg/m^3^ Hg vapor group was precluded because exposure chamber space and exposure durations limited the number of groups per experiment.

a Group included in both experiments.

**Table 2:** Number of litters, number of pups per litter, and body weight (mean ± SD) on PND1 and PND4 in experiment 1.

	Group [MeHg (ppm) × Hg vapor (μg/m^3^)]							
	0 × 0	3 × 0	6 × 0	0 × 300	3 × 300	0 × 1,000	3 × 1,000	6 × 1,000
No. of litters								
PND1	14	10	10	12	10	10	9	11
PND4	14	10	9	12	10	10	9	11

No. of pups per litter								
PND1	10.8	11.7	8.4	11.8	11.9	10.7	10.4	11.1
PND4	10.8	11.6	9.2	11.8	11.5	9.3	10.1	10.7

Body weight (g)								
Males								
PND1	6.40 ± 0.07	6.34 ± 0.11	6.61 ± 0.14	6.21 ± 0.16	6.10 ± 0.16	6.47 ± 0.14	6.32 ± 0.11	6.48 ± 0.21
PND4	9.44 ± 0.80	9.11 ± 0.96	9.85 ± 1.24	9.07 ± 1.27	8.92 ± 0.85	8.94 ± 1.17	9.09 ± 1.19	9.55 ± 1.72
Females								
PND1	6.11 ± 0.10	6.06 ± 0.09	6.10 ± 0.11	5.92 ± 0.13	5.82 ± 0.17	6.11 ± 0.11	6.01 ± 0.08	6.19 ± 0.16
PND4	9.02 ± 0.67	8.66 ± 1.03	9.36 ± 1.16	8.65 ± 1.11	8.52 ± 1.04	8.38 ± 1.61	8.45 ± 1.32	9.10 ± 1.41

Group means were calculated using the mean litter weight per group per day. Body weight on PND1 did not differ among groups.

**Table 3:** Number of litters, number of pups per litter, and body weight (mean ± SD) on PND1 and PND4 in experiment 2.

	Group [MeHg (ppm) × Hg vapor (μg/m^3^)]							
	0 × 0	3 × 0	6 × 0	9 × 0	0 × 1,000	3 × 1,000	6 × 1,000	9 × 1,000
No. of litters								
PND1	10	11	7	11	10	10	8	11
PND4	10	11	7	7	10	9	8	9

No. of pups per litter								
PND1	9.4	10.7	8.1	10.9	11.4	10.2	11.3	8.1
PND4	9.2	10.6	7.4	12.6	10.3	11.1	7.8	6.8

Body weight (g)								
Males								
PND1	6.42 ± 0.56	6.28 ± 0.26	6.53 ± 0.52	5.89 ± 0.64	6.05 ± 0.42	6.22 ± 0.78	5.96 ± 0.49	6.07 ± 0.65
PND4	9.03 ± 1.37	8.87 ± 0.54	9.15 ± 0.85	7.33 ± 0.58	8.58 ± 1.47	9.18 ± 0.80	8.20 ± 1.97	8.20 ± 1.58
Females								
PND1	6.05 ± 0.46	5.94 ± 0.25	6.10 ± 0.62	5.59 ± 0.62	5.73 ± 0.46	6.07 ± 0.42	5.69 ± 0.78	5.71 ± 0.55
PND4	8.73 ± 1.17	8.40 ± 0.60	8.45 ± 1.20	7.17 ± 0.92	8.12 ± 1.32	8.47 ± 0.93	7.47 ± 2.60	7.06 ± 1.53

Group means were calculated using the mean litter weight per group per day. More pups were lost in the 9-ppm MeHg groups with or without Hg vapor exposure between PND1 and PND4. Body weight on PND1 did not differ among groups. There was a main effect of MeHg on body weight on PND4 (*p* = 0.0203 for male and *p* = 0.0055 for female).

**Table 4:** Total, inorganic, and organic Hg concentrations (mean ± SD) in pup brain on PND4 in experiment 1.

	Group [MeHg (ppm) × Hg vapor (μg/m )]							
	0 × 0	3 × 0	6 × 0	0 × 300	3 × 300	0 × 1,000	3 × 1,000	6 × 1,000
Total Hg (ng/g)								
Males	20 ± 7	2,773 ± 231	8,997 ± 2,949	42 ± 13	2,946 ± 208	50 ± 11	3,244 ± 626	8,064 ± 1,524
Females	26 ± 15	2,723 ± 86	9,262 ± 2,810	43 ± 7	2,928 ± 383	51 ± 14	2,685 ± 571	7,900 ± 2,150
Inorganic Hg (ng/g)								
Males	5 ± 0	140 ± 23	490 ± 209	11 ± 5	150 ± 35	18 ± 5	172 ± 37	350 ± 113
Females	7 ± 3	140 ± 15	505 ± 169	13 ± 6	148 ± 30	20 ± 3	151 ± 32	290 ± 149
Organic Hg (ng/g)								
Males	15 ± 7	2,632 ± 231	8,507 ± 2,749	31 ± 13	2,796 ± 180	33 ± 7	3,072 ± 593	7,715 ± 1,420
Females	20 ± 12	2,583 ± 101	8,757 ± 2,658	31 ± 4	2,780 ± 359	32 ± 12	2,534 ± 539	7,510 ± 2002

**Table 5:** Total, inorganic, and organic Hg concentrations (mean ± SD) in pup brain on PND4 in experiment 2.

	Group [MeHg (ppm) × Hg vapor (μg/m^3^)]							
	0 × 0	3 × 0	6 × 0	9 × 0	0 × 1,000	3 × 1,000	6 × 1,000	9 × 1,000
Total Hg (ng/g)								
Males	26 ± 13	3,469 ± 254	8,254 ± 1,078	16,122 ± 4,419	32 ± 8	3,286 ± 321	6,796 ± 1,684	11,795 ± 3,156
Females	29 ± 16	3,703 ± 72	9,703 ± 2,216	16,545 ± 5,941	33 ± 11	3,379 ± 287	6,468 ± 1,727	13,177 ± 5,007
Inorganic Hg (ng/g)								
Males	8 ± 6	167 ± 19	452 ± 95	612 ± 52	7 ± 3	160 ± 48	370 ± 75	518 ± 107
Females	8 ± 7	165 ± 18	475 ± 83	678 ± 151	5 ± 0	156 ± 22	366 ± 58	668 ± 203
Organic Hg (ng/g)								
Males	18 ± 11	3,302 ± 240	7,803 ± 1,004	15,511 ± 4,376	25 ± 7	3,127 ± 293	6,426 ± 1,622	11,277 ± 3,052
Females	21 ± 15	3,538 ± 62	9,228 ± 2,150	15,867 ± 5,811	28 ± 11	3,223 ± 276	6,102 ± 1,670	12,509 ± 4,812
