# Correction: Organic and Inorganic Mercury in Neonatal Rat Brain after Prenatal Exposure to Methylmercury and Mercury Vapor

**DOI:** 10.1289/ehp.0900956-erratum

**Published:** 2009-09-29

In the manuscript originally published online, right-hand columns showing higher Hg vapor doses were inadvertently omitted from Tables 2–5, error bars were omitted from Figure 1A and 1B, and Figure 2B was incorrect. The tables and figures have been corrected here.

